# Human security as biosecurity: Reconceptualizing national security threats in the time of COVID-19

**DOI:** 10.1017/pls.2021.1

**Published:** 2021-01-19

**Authors:** Craig Albert, Amado Baez, Joshua Rutland

**Affiliations:** 1 Augusta University; 2 Augusta University; 3 Augusta University

**Keywords:** Human Security, Biosecurity, Health Security, National Security, COVID-19

## Abstract

Research within security studies has struggled to determine whether infectious disease (ID) represents an existential threat to national and international security. With the emergence of SARS-CoV-2 (COVID-19), it is imperative to reexamine the relationship between ID and global security. This article addresses the specific threat to security from COVID-19, asking, “Is COVID-19 a threat to national and international security?” To investigate this question, this article uses two theoretical approaches: human security and biosecurity. It argues that COVID-19 is a threat to global security by the ontological crisis posed to individuals through human security theory and through high politics, as evidenced by biosecurity. By viewing security threats through the lens of the individual and the state, it becomes clear that ID should be considered an international security threat. This article examines the relevant literature and applies the theoretical framework to a case study analysis focused on the United States.

The spread of infectious disease (ID) in catastrophic proportions, such as in endemics and pandemics, is a threat to national and international security. In fact, the threat to human security and biosecurity should be included along with other perceived security threats such as conventional warfare and terrorism. Unlike ISIS, al-Qaeda, and the Taliban, ID has no natural enemy; it cannot be stopped by policy, borders, or alliances. Yet it gets less treatment in the literature and by policy wonks than do more traditional security threats. In 2006, Elbe noted that HIV/AIDS was a global security threat, evincing that the daily death toll from the disease was three times higher than the number who perished on September 11, 2001. It was not until the Bill Clinton administration that HIV/AIDS was taken seriously as an international security threat by policymakers and scholars alike.

As Elbe (2006) notes, the major shift to this treatment of a disease as a security threat did not happen until 2000. On January 10 of that year, the United Nations (UN) Security Council officially declared HIV/AIDS a threat to international peace and security in Africa. This was a remarkable moment for the nexus between international security and ID, as this was the first time an ID was declared such a threat. And, as Elbe elaborates, the declaration was accompanied by the release of the National Intelligence Estimate announcing the Clinton administration’s official designation of HIV/AIDS as a national security threat to the United States.

As of October 2020, the global daily death toll for severe acute respiratory syndrome coronavirus 2 (SARS-CoV-2)—hereafter referred to as COVID-19—consistently ranged between 4,000 and 6,000 people, with a total global death toll surpassing one million. The U.S. daily death rate varied from 259 to 928 in the last week of September 2020 alone, with the total number of dead at just over 200,000.^1^ Thus, worldwide, the daily average death rate surpasses the loss of life on September 11. In the United States alone, the numbers exceed the loss of life from September 11 each week. Even considering the extant literature on ID as an international security threat, and efforts by past U.S. presidential administrations and the UN and other international organizations, in some corners of the international arena, including policy and academic circles, the threat seems to have been unheeded. Today, given the statistics on COVID-19, there is a great debate as to whether ID should be considered a threat to national and international security.

Perhaps prophetically, Evans (2010) emphasized the need to shift from a view focusing solely on international warfare as a threat to national and international security toward one including ID: “National security must be redefined for a new era where conventional war is no longer the primary physical threat to a state; instead, the focus must shift to include threats from disease that challenge the interests of the United States abroad, and the safety of its citizens at home” (p. 100). Adding substantive evidence to the dilemma, Singh (2019) notes that 34% of all deaths worldwide are attributable to ID, while deaths from war and terrorism account for only 0.64% (p. 15). The unknowns of COVID-19 make any future analysis concerning its long-range breadth and scope troubling to assess and create massive uncertainty in projecting societal, economic, and political consequences (Heisbourg, 2020, p. 8).

This article examines to what extent and in what ways COVID-19 represents a challenge to global security. It seeks to address the question, “Is COVID-19 a threat to national and international security?” Although the security threat posed to states and the international system by ID has been analyzed in the literature, there is no consensus as to whether these threats should be treated within the same paradigm as “high politics,” such as conventional war and terrorism, or whether ID should be relegated to the realm of “low politics,” along with such phenomena as global climate change, immigration crises, and international drug trafficking (Baldwin, 1997). This article argues that when viewed through the prisms of human security theory and biosecurity, it becomes clear that COVID-19 is a threat to national security in the context of high politics. This thesis is evinced by the sheer numbers of individuals infected and killed, national economic losses, military preparedness and readiness, and the effects of the disease on the national health infrastructure system and public trust. Additionally, this article demonstrates the potential threat that COVID-19 poses to international security by examining the possible effects on the geopolitical landscape and the perceived windows of opportunity and vulnerabilities that strategic adversaries may take advantage of because of the pandemic’s existence: opportunities that would not exist without COVID-19’s presence.

This article adds to the literature in two main ways, making clear its importance to the literature on ID and international security. First, the theoretical literature on the subject is rich, and policy documents abound on the nexus between ID and security. However, as President Donald Trump’s administration made clear in disbanding previously established pandemic response protocols, this literature has gone unheeded in some circles. Additionally, several state actors, particularly but not exclusively China, have maintained outdated regulations with poor health standards in some of their environments that are most susceptible to infection and spread of ID. Even though great strides have been made, the connection between ID and security is not fully grasped. Also, amid the increase in serious scholarly work within international relations and health security, there has also been a rise in so-called anti-science and anti-vax movements, which are largely grounded in conspiracy theory, crippling international governing bodies such as the World Health Organization (WHO). Thus, the connection between ID and national and international security warrant reemphasis and revisiting. This article serves this purpose.

Second, the literature seems to be split, as explained later, between those academics fitting somewhere in the realm of critical theory, who largely agree with classifying ID as a clear threat to security, and more “hard power” and “high politics” scholars, who argue that only conventional war and great power politics should be considered serious security threats. This article emphasizes an original point: regardless of one’s theoretical stance, within human security theory (a more critical approach), and within biosecurity and the typical realist theoretical-speak of hard power and high politics, ID, with an emphasis on COVID-19, clearly represents a threat to security across the theoretical spectrum. Thus, this article serves to solidify the previously made arguments, but often unheeded, that ID represents a clear and present danger to national and international security.

We proceed in four sections. First, the two main theoretical paradigms used to analyze ID are discussed in detail: human security theory and biosecurity. Then, the article illustrates the medical trajectory and contagion rates and provides a brief case study of the effects of COVID-19. Next, we provide a brief illustrative case study of events in which COVID-19 has wreaked havoc, focusing mainly on the United States but also, when possible, using comparative evidence as well. This section evinces the areas in which COVID-19 represents a threat to national and international security according to the theoretical prisms provided in the first section. Therefore, this article is original in that it not only seeks to solidify the theoretical ground and help resolve the debate of the connection between ID to international security, but also serves as a clear and illustrative application of a disease to demonstrate precisely how and in what ways it is a threat to security. Lastly, this article briefly outlines some policy recommendations concerning the nexus between infectious disease and national security. In summary, COVID-19 as a national security threat when viewed at the nexus of human security theory and biosecurity will be discussed in depth throughout.

## Human security theory and biosecurity

Traditionally within security studies, only “high politics” or existential threats to state capacity from war and terrorism have been seriously studied. Public health and ID have usually been studied through “low politics,” existing alongside serious issues but ones not considered immediate existential threats, including climate change, world hunger, and energy politics. Baldwin (1997) explains the differences between these two “politics” deftly: “If military force was relevant to an issue, it was considered a security issue [high politics]; and if military force was not relevant, that issue was consigned to the category of low politics” (p. 9). According to Baldwin’s explanation, if it involves force, it exists within the realm of high politics. If there is no use of force, then the contested issue exists within low politics. Buzan et al. (1998) argue that security is about survival: “It is when an issue is presented as posing an existential threat to a designated referent object (traditionally, but not necessarily, the state, incorporating government, territory, and society)” (p. 21). Low politics incorporates everything else. As Youde (2016) writes, “Low politics are then those issues less existentially vital to the state and motivate state action only when the higher issues are adequately addressed” (p. 158).

Low-politics issues, then, are only dealt with or discussed once threats to a state’s security have been adequately safeguarded. Only when a terrorist threat is eliminated, for example, is that country free to consider domestic homelessness. Within this understanding of high politics, “Once a state is able to safeguard its military, territorial, and political interests from outside threats, it is perceived as having attained national security” (Iqbal, 2006, p. 632). High politics or military issues that threaten the state, or the sovereignty of the country, are understood as national security or within the national interests; when considering power politics, or the relationship between national security and interdependence, the issue at stake involves international security. Or, as Newman (2001) states, “International security has traditionally been defined as military defense of territory” (p. 241).

The problem that separating high and low politics creates is that academics and policymakers spend more time researching and solving issues of high politics without giving much concern to matters existing in low-politics zones. The rationale behind this is straightforward: immediate existential threats must be resolved before a state can ameliorate less immediate risks. Threats to international security are the most serious because they involve the most severe consequences on the international stage and threaten to destabilize the system. However, once a concept that usually exists in the low-politics realm emerges as a serious existential threat, such as pandemic diseases, policies and immediate infrastructure relief to handle this threat are slow to form (Enemark, 2009). Global health and ID have been examined in this context of low politics or nonexistential security discourses. The global health crises of HIV/AIDS and H1N1 prompted more attention to be given to this area, so much so that the United States officially designated pandemics as a national security threat at the end of the 1990s (Elbe, 2010). Still, there is considerable debate as to the benefits of the high/low politics dichotomy and of even understanding ID and health within this framework. However, it is a useful point of departure for examining whether a particular ID merits consideration within the scope of security studies.

This section briefly illustrates two types of security frameworks to help the reader investigate how ID—in this case, COVID-19—threatens national and international security. The two areas explored are human security theory and biosecurity. In both of these areas, COVID-19 poses an existential threat and should be taken seriously within national and international security. These sections reify and add to the literature on how and in what ways ID are threats to national and international security. Based on the theory provided, this article will apply the case of COVID-19 using these paradigms as premises to clearly denote the threat that ID poses to security and how international relations more broadly can account for this type of nontraditional security threat.

### Human security theory

Perhaps more than any theoretical framework, human security is best equipped to analyze and assess the impact that ID has on national security. As Peterson (2002) notes, most scholars who study the nexus between disease and security do so from the human security tradition, which seeks to expand security beyond the state to include basic human needs such as health (p. 44). In opposition to traditional notions of national security, human security focuses more on the individual’s or group’s well-being and welfare (Iqbal, 2006). National security, on the other hand, is focused on the well-being of the state or the state’s survival. The human security paradigm argues that there are many more complex and varied threats to one’s existence other than direct physical violence. These should be included in conversations about state security (Iqbal, 2006). Although admittedly vague, the conceptualization of human security, broadly defined as an individual’s freedom from want and freedom from fear, provides a useful comparison with national security’s focus on the survival of the state. Accordingly, state security must be complemented by other elements, including human rights and public health; it must focus on everyday people, rather than on the perpetuation of the state only (Iqbal, 2006).

Elements counted as existential threats within the human security literature include economic security, political security, access to food and health care, personal and community safety, and environmental security (Iqbal, 2006). Whereas national security has the imperative of defending territory against external threats, human security recognizes that, globally, there are significant threats to security emanating from disease, hunger, pollution, crime, and domestic violence, to name a few (Newman, 2001, p. 241). Viewed in this manner, the foundational premise of human security is the orientation of all facets of security around and in the interest of the individual (Newman, 2001, p. 243). Conceptualizing human security has been contentious in the literature, and varied understandings have been presented. The unifying elements include a shift to the individual as the referent object of security and the idea that a threat to the quality of life of individuals should be considered a threat to security (McDonald, 2002, p. 279). It should be emphasized that human security does not ignore state security, or what is traditionally regarded as military or national security; instead, human security treats national security as no more than coeval to individual security (Bajpai, 2003, p. 217).

A prioritized concern for human security is public health. This notion “entails people’s ability to maintain a quality of life that does not fall below the level at which they feel secure” (Iqbal, 2006, p. 633). Within the public health sector concerning human security, preventing the spread of ID, or finding cures to help protect the individual, has been a focus for decades. Human security and health security have been priorities for the WHO at least since 1993, when it released data on a global emergency status about the need for increased vigilance against lethal viruses attacking the nation-state and spreading quickly as a result of globalization (Pugu & Buiney, 2017, p. 32). Accordingly, pandemics and diseases have become securitized in the academic literature and within policy circles. Securitization refers to the process of taking an issue out of its benign status in nonsecurity debates and elevating it to the security sphere by portraying it as a threat to security (Elbe, 2006, p. 126). This securitization happened to the issue of HIV/AIDS, where officials increasingly argued that the disease posed a threat to the survival of communities, states, and militaries unless drastic measures were taken by national and international actors to better address the global pandemic.

This response is appropriate given the substantial proliferation of HIV/AIDS across the developing world and its eventual spread to more modernized countries. In the mid- to late 1990s, HIV/AIDS rocketed to prominence within the UN’s agenda, taking second place in priority only to military interventionism and peacekeeping missions (Boone & Batsell, 2001). However, political science as a discipline was slow to react, initially dismissing the HIV/AIDS issue as “too private, too biological, too microlevel and sociological, too behavioral and too cultural” for discussion in the context of politics or international relations (Boone & Batsell, 2001, p. 4). Yet the political impact of the HIV/AIDS pandemic posed very real and, potentially, impactful consequences for the global community as states grappled with the lethality and propensity of the disease to spread, regardless of borders or sovereignty (Boone & Batsell, 2001). HIV/AIDS still represents “one of the gravest threats to public health and development” in sub-Saharan Africa, yet it took more than two decades for the disease to be considered a security issue (Boone & Batsell, 2001).

This slow and at times lackluster response to pandemic threats on the part of the political science field remains a troublingly persistent trend. Discussing the nontraditional threat to health security, Elbe (2011) notes that “the core assertion of the idea of health security is that insecurity is no longer caused solely by the military capabilities and hostile political intentions of other states; it is similarly brought about today by the presence and rapid circulation of disease within and across populations” (p. 849). Davies contextualizes the threat from ID by outlining the recent discovery of new pathogens. She notes that since the 1980s, the rate of discovery of new diseases has been at least one per year. Davies (2008) argues that discoveries and the outbreak of severe acute respiratory syndrome (SARS) and the H5N1 strain of avian influenza “led to the argument that the world cannot escape a potential epidemic influenza that could kill anywhere between 2 million and 12 million people. These developments have all served to increase calls for infectious diseases to be targeted as threats to national security” (p. 298). Commenting on the security threat presented by SARS, Prescott (2003) notes that “accordingly, developed countries must accept that they are only as secure as the world’s weakest public health system and for as long as it takes a passenger to travel from that location” (p. 213).

Price-Smith’s *Contagion and Chaos* (2008) supports the notion that biological threats represent a threat to human security worthy of high politics. However, his findings indicate a historical lack of commitment to considering these threats with such gravity. Price-Smith (2008) argues that despite the “significant levels of fear and psychological trauma [generated] in the effected populations,” impedance of international trade and migration flows, and the “minor to moderate economic damage to the economies of affected Pacific Rim countries (particularly China and Canada),” the resulting policy adjustments were lackluster (p. 139). Domestically, moderate institutional changes occurred, but purely “ephemeral change” was observed at the global level (Price-Smith, 2008, p. 139). The greatest impact of the disease at the global level was the revision of the International Health Regulations and an update of the list of reportable diseases (Price-Smith, 2008, p. 156). While the WHO enjoyed a temporary increase in power during the outbreak, it was ultimately *temporary*, with sovereign states displaying no indication of increased willingness to comply with “international health regimes” (Price-Smith, 2008, p. 156).

Price-Smith (2008) further recognizes the potential of SARS and similar viruses, such as COVID-19, to become “a global pandemic that might have generated much greater loss of life and economic damage,” constituting a threat to global security (p. 139). He argues that the uniquely significant domestic responses to SARS compared with HIV, malaria, and tuberculosis was largely due to a few specific aspects of SARS not observed in other diseases. Namely, SARS was novel, exhibited high virulence and transmissibility, presented an immediate and unavoidable socioeconomic crisis for policymakers at all levels, and represented an “exogenous shock” that threatened the material interests of global elites (Price-Smith, 2008, pp. 156). COVID-19, a virus sharing many of the same traits as SARS, has obviously demonstrated Price-Smith’s point by surpassing the effects of SARS in each of these areas.

Davies (2013) analyzes the 2005 revising of the International Health Regulations following the SARS outbreak and the aftermath of these revisions. In essence, the alterations to the regulations were designed to attach health to security in order to highlight the regulations’ importance and to “help sustain the political will needed to achieve core capacities,” or baseline measurements of preparedness, by July of 2012 (Davies, 2013, p. 21). The core areas of interest deemed necessary for proper pandemic preparedness included national legislation, policy and financing, coordination and National Focal Point communications, surveillance, response, preparedness, risk communication, and human resources and laboratories. However, the shockingly high number of states that failed to meet these guidelines (110 of 195) became cause for alarm in 2013, as this indicated the possibility that these states either were not taking the commitment to preparedness seriously or were facing immense challenges to establishing core capacities (Davies, 2013).

Davies (2013) found that it was more likely that these 110 states were facing immense difficulties in establishing these core capacities. Many of these states did not appear to espouse political objections to bettering health systems but were trying to fix health systems that suffered from fragmented, underfunded, and understaffed health systems. However, this poses a major problem, as capacity failures by any one state in a globalized world could mean disaster for the entire global system (Davies, 2013). COVID-19 has demonstrated this in spades. Davies (2013) addresses this issue and suggests three strategies for implementation moving forward to foster better pandemic preparedness. The first strategy involves promoting the association of health policy commitments and security to raise the priority of health security and to deliver better results. The second involves promoting universal national health care systems that are accessible and equitable, as these systems are widely recognized as essential for effective pandemic prevention and response. The third asserts that regulation compliance must be approached from a regional perspective that remains considerate of regional context and norms. These efforts must be supported by global institutions such as the Peacebuilding Commission, United Nations Development Programme, and United Nations Children’s Fund (Davies, 2013, p. 24).

Despite past global experience with ID, the COVID-19 crisis has deviated from the traditional international relations method of addressing “problems without passports” (Johnson, 2020, p. 1). Whereas governments would normally rely on international organizations such as the WHO to manage a global response, backlash against these organizations has been on full display. They have been the subject of blame and animosity from several states as divisive reactions to experts flourished, while some have prioritized narrow and short-sighted interests (Johnson, 2020). Johnson (2020) concludes that the tumultuous first few months of the crisis are an example of how the world behaves without resolute leadership to overcome common versions to international organizations typically sparked by concerns over sovereignty or anti-elitism. This unusual crisis response should be cause for concern. Historical pandemic responses, such as the responses to HIV/AIDS and SARS, were indicative of cracks in the complex globalized system (Benatar, 2002). The initial months of the global response to COVID-19 may indicate that those cracks have deepened.

Specifically situating COVID-19 as a threat to human security, Milani (2020) argues, “A compelling novelty of this pandemic is the worldwide anthropological experience of fear and death in such a short span of time. COVID-19 has expanded as a security threat that is existential in scope.” The scholar continues, “The threat has reached individuals in direct, palpable and conspicuous ways. It has touched everyone’s [neighborhood], families and many households” (p. 144). Human security is the idea that the individual must be protected from harm and free from want. This article illustrates how COVID-19 threatens human security and should be considered a threat to national and international security. However, as is shown in the next section, COVID-19 is also a threat from the prism of biosecurity.

### Biosecurity

Biosecurity is a nebulous term that is hard to conceptualize.^2^ The concept is usually grouped with bioterrorism and biosafety, but it is quite distinct from these and often includes measures to prevent bioterrorism and maintain biosafety measures. For comparison’s sake, bioterrorism is understood as “the deliberate use of biological warfare agents (BWAs) that can kill or incapacitate living being[s]. These BWAs are also called weapons of mass destruction (WMD) that targets lives without affecting infrastructure” (Dhaked, 2017, para. 1). Agents used in bioterrorism include bacteria, viruses, and toxins (Dhaked, 2017). Often, biosecurity and biosafety are merged into one definition, such as, “In a broad sense, biosafety, and biosecurity … refer to a nation’s ability to respond effectively to biological threats and related factors” (Zhou et al., 2019, p. 15). Within this meaning, biosecurity and biosafety include safeguarding against and mitigating emerging infectious diseases; protection from biological warfare and bioterrorism; the prevention of malicious biotechnology misuse; the insurance of laboratory biosafety; the defense and securitization of special biological resources; and “the prevention of invasion by alien organisms” (Zhou et al., 2019, p. 15). Biosafety has a narrow definition as well. It is referred to as “keeping laboratory workers, the community, and the environment safe” (Gronvall, 2017, p. 25). For the present purposes, only biosecurity in a nonlaboratory setting is considered. As such, biosecurity has wide-ranging meanings.

Tucker (2006) defines the term “biosecurity” narrowly as the “measures to prevent the theft, diversion, and deliberate misuse of disease agents” (p. 120). Koblentz (2012) conceptualizes President Barack Obama’s biosecurity strategy as using a broad understanding: it encompasses “the full range of biological risks to humans, plants and animals, ranging from naturally occurring infectious diseases through laboratory accidents and dual-use research to disease outbreaks deliberately caused by states or terrorist” (p. 133). Gostin and Fidler (2006–2007) argue that biosecurity is “society’s collective responsibility to safeguard the population from dangers presented by pathogenic microbes—whether naturally occurring or intentionally released” (p. 438). As such, Gostin and Fidler’s concept incorporates threats presented by biological weapons and naturally occurring disease epidemics. They note that not all diseases represent a threat to national security and thus do not fall within the realm of biosecurity. Gostin and Fidler (2006–2007) argue that to be a concern within the realm of biosecurity, a natural or intentionally released epidemic must have the potential to disrupt the normal functioning of societies (p. 438). They also note that communicable diseases represent one of the most significant burdens of morbidity and mortality globally (Gostin & Fidler, 2006–2007, p. 442). Biosecurity thus emerges at the convergence of security and public health.

The convergence of public health and national security has been an increasing topic of discussion within the security studies literature. Enemark (2009) argues that a particular disease may be a security issue when its effects threaten to impose an intolerable burden on society (p. 191). She elaborates, “The essence of the global public-health challenge posed by pandemic influenza would thus be scarcity of resources. … The human and material resources for health care, which are usually stretched even in ordinary times, would be rapidly overwhelmed” (Enemark, 2009, p. 194). The massive scale of the threat is what makes ID a national security threat within the biosecurity paradigm. It becomes a threat in terms of morbidity, mortality, and the perceived fear of infection and overwhelming the public health infrastructure. The importance of biosecurity and its impact on public health has managed to create a paradigm existing within both the fields of biosecurity and human security: health security. Concerning the specific threat to health systems, health infrastructure, and health itself, Dinicu (2020) discusses the impact of health security as an issue that can threaten or challenge national security. The scholar notes that health security is a citizen’s right and a primary area of action to preserve national security. In this light, if a problem challenges public health, it is at the same time a challenge to national and international security (Dinicu, 2020).

The 2019 Worldwide Threat Assessment of the U.S. Intelligence Community provides a specific example of the existential threat pandemics pose to national and international security at the nexus of bio and human security. Daniel R. Coats (2019), former director of national intelligence, noted, “We assess that the United States and the world will remain vulnerable to the next flu pandemic or large-scale outbreak of a contagious disease that could lead to massive rates of death and disability, severely affect the world economy, strain international resources, and increase calls on the United States for support” (p. 21). A 2016 article published in the *New England Journal of Medicine* announced that the lack of attention spent both nationally and internationally on ID preparedness was a neglected dimension of security:

Infectious disease represents one of the most potent risks facing humankind. Few events could cause such loss of life and damage to livelihoods. Yet the global community spends relatively little in protecting humankind from the threat of pandemics. As compared with our position vis-à-vis other threat to human and economic security, such as war, terrorism, nuclear disaster, and financial crises, we are underinvested and underprepared. Pandemics are the neglected dimension of global security. (Sands et al., 2016, p. 1287)

Additionally, the emphasis placed on influenza pandemic preparedness demonstrates the impact ID has on national security. For instance, The 2017 update of the Pandemic Influenza Plan from the U.S. Department of Health and Human Services indicated that worldwide planning for a novel influenza pandemic had helped national security: “All of these international activities serve to directly improve national security as they enable rapid communications, surveillance, and mitigation of emerging novel influenza viruses with other countries to ensure a better national response” (p. 9). Furthermore, the National Security Strategy of the United States mentions the threat from ID explicitly and notes that they demonstrate, “the impact of biological threats on national security by taking lives, generating economic losses, and contributing to a loss of confidence in government institutions” (White House, 2017). And, as Chang and Hsu (2017) write, “Protecting [the] population from biological threats is not only indispensable, but morally non-negotiable” (p. 2).

It is clear from this assessment that health security, as a concept bridging the nexus between human and biosecurity, is of direct importance to national security. But how exactly is ID an immediate threat to human and biosecurity? Additionally, how does COVID-19 fit within this framework of national and international security, and in what specific ways does it threaten security? Examining the particular ways COVID-19 threatens both human security and biosecurity, including health security, and threatens the global security environment, is imperative.

## Understanding SARS-CoV-2

Coronaviruses are pathogens that affect humans and other animals. Currently, seven major types of human coronaviruses are known, including COVID-19 (Friedman et al., 2018). They can infect a variety of hosts, including humans, through “respiratory, gastrointestinal, hepatic, and central nervous systems” (Chen et al., 2020, p. 418). Outbreaks of coronaviruses and similar respiratory infections, such as SARS in 2002 and Middle East respiratory syndrome (MERS) in 2012, have become increasingly common. They are unlikely to decrease in frequency or severity going forward (Chen et al., 2020). Therefore, Chen et al. (2020, p.418) argue that “there is an urgent need to develop effective therapies and vaccines against [coronaviruses].”

In late 2019, a coronavirus that has now been named severe acute respiratory syndrome coronavirus 2 (SARS-CoV-2) affected the city of Wuhan, China, developing into an epidemic that quickly spread to other states—first to South Korea, Europe, and the Middle East—and eventually across the globe with remarkable speed (Freedman, 2020). In February 2020, the WHO named this new disease coronavirus disease 2019, or COVID-19 (Cevik et al., 2020, p. 842). Since December 2019, COVID-19 has extended to all continents, and the WHO declared it a public health emergency in January 2020. It was later classified as a pandemic in March 2020, and as of late September 2020, COVID-19 had spread to nearly 30 million confirmed cases worldwide (Dong et al., 2020). Overcoming the virus was an existential issue. As Freedman (2020, p. 25) notes, “Its virulence and lethality meant that its effects were of a different order than the normal seasonal flu, to which it was often inappropriately compared.” The symptoms of COVID-19 can range from mild to severe. Fever, cough, and shortness of breath are the most common symptoms. Coronavirus can also cause “sudden onset of flu-like symptoms,” including “fever and dry cough,” as well as myalgia, headache, and “chills/rigors” (Friedman et al., 2018). Additionally, gastrointestinal symptoms such as nausea, vomiting, and diarrhea have been observed in some COVID-19 patients (Nobel et al., 2020). Patients who contract COVID-19 may worsen rapidly and die of multiple organ failure. Person-to-person transmission is the primary means of SARS-CoV-2 transmission, and it is now known to spread via aerosolized respiratory droplets.

### COVID-19 epidemiology and public health

COVID-19 patients typically begin to exhibit symptoms after an incubation period of 4.2 days on average (95% CI [3.5, 5.1]) (Sanche et al., 2020). It is now estimated that approximately 80% of patients who become infected with COVID-19 will present with mild or moderate symptoms. Around 15% will present with a severity of symptomatology that will require hospitalization (Wiersinga et al., 2020, p. 784). The remaining 5% will present with a critical form of the disease. Hospitalization seems to occur, on average, around 5.5 days (95% CI [4.6, 6.6]) after the onset of symptoms (Sanche et al., 2020). Sufficiently advanced health care systems may have the ability to heal some of these critical patients, but even the most advanced health systems can become overwhelmed by the sheer amount of people in need of hospitalization. It has been determined that COVID-19 harshly affects the elderly and those with preexisting medical conditions (Cevik et al., 2020, p. 842). The pediatric population seems to be less affected by the disease. Implementing certain measures, such as isolation, quarantine, and social distancing, to limit the spread of the virus has been critical in reducing the spread of the disease and has led to a reduction of new cases and critical cases and decreased utilization of health facilities (Wiersinga et al., 2020, p. 788).

Epidemiology is critical in the surveillance of disease, allowing the collection of data to calculate incidence, prevalence, hospitalizations, and deaths. These numbers will vary by region and are influenced in the long term by systemic factors such as the amount of testing, health care quality, availability of treatment, length of time since the initial outbreak, and other more individually specific characteristics such as age, sex, and general health of the collective population (Paulino-Ramirez et al., 2020). Other measures include the case fatality rate (CFR), which estimates the proportion of deaths among identified and confirmed cases, and the infection fatality rate (IFR), which estimates the death rate among all infected individuals, including unconfirmed cases (World Health Organization, 2020). The CFR of COVID-19 is estimated at approximately 2%, compared with 10% for SARS, 34% for MERS (Mahase, 2020), and 2.5% for the 1918 influenza pandemic (Wong et al., 2013). However, COVID-19’s CFR is somewhat deceptive, as its high propensity for infection has resulted in a higher death count than SARS and MERS combined (Mahase, 2020). The IFR for COVID-19 has been estimated at 1.3%, a rate significantly higher than the estimated rate for seasonal influenza (roughly 0.1%) (Basu, 2020).

The effective reproduction number (R) provides a more practical parameter than the basic reproduction number (R_0_) for the characterization of ID epidemics. This is because it accounts for the presence of immune individuals, which R_0_ does not consider. However, R_0_ numbers are more readily available in the current literature. Liu et al. (2020) found that for COVID-19, the average R_0_ is 3.28, with a median of 2.79, which is comparable to the average range of 2 to 5 for SARS but considerably higher than the WHO-estimated R_0_ of 1.95. Periodic reassessment of an ID’s R number can help guide public health strategies during long-standing epidemics such as COVID-19 (Pan et al., 2020, p. 1918).

### Dispersion parameter K versus R

K is a statistical tool that helps explain the variation in the reproduction number (R). Both R and K are necessary tools for understanding the spread of COVID-19. Pathogens have unique ways of spreading, which statisticians describe by using the dispersion parameter (K) to detail the variability of the infection (Anzai et al., 2020). K describes the level of variation within a distribution. Generally, the smaller the K value is, the more transmission can be expected to originate from a small number of infectious people. However, some infectious individuals might generate a high number of secondary cases, while others may not generate many secondary cases at all (Hartfield & Alizon, 2020)

Once the K value exceeds 10, it signifies that most infected individuals are generating similar numbers of secondary cases, rather than large case numbers coming from isolated super-spreading events. Once K drops below 1, however, the potential for super-spreading exists. Smaller values of K thus mean that one infected person can cause many new cases within a short time frame (Zhang et al., 2020). Anzai et al. (2020) estimate the K value of COVID-19 to be 0.54, while Zhang et al. (2020) estimate that its K value may be as low as 0.25. The consensus remains that the K value is low, which indicates a high probability of super-spreading. This is consistent with the high number of super-spreader events observed during the COVID-19 pandemic (Zhang et al., 2020). The K value is particularly critical in the late stages of the epidemic when the virus is nearly eradicated. The presence of a low K value indicates the possibility of a quick rebound of the epidemic. Outbreaks further demonstrate how damaging super-spreading events can be (Kupferschmidt, 2020). Epidemiology efforts thus need to diligently identify the risks associated with social and economic reopening. Methods of identifying and tracking potential super-spreaders is fundamental for the prevention of future outbreaks and statistics regarding the K value can assist in this important process.

From a practical epidemiological standpoint, analysis of CFR, R_0,_ K, and positivity rates and trends can be used for strategic and operational planning and country to country benchmarking. These numbers also quite accurately assess whether a disease is a threat to individuals and society or the nation-state as a collective. According to the rates, and based on the conceptualizations of human security theory and biosecurity, the current article argues that these numbers are high enough to warrant considering COVID-19 a threat to national and international security. Outside of these numbers, however, multiple variables help visualize how and why COVID-19 is a threat to national security. Beyond a doubt, the disease touches on many threat vectors and may endanger military preparedness, health infrastructure, national and international economic security, and more. The following section briefly explains some of the most threatened variables to consider according to human security and biosecurity. It should become clear that, according to the literature and the evidence provided, COVID-19 is an existential threat to security.

## COVID-19 as a threat to national and international security

COVID-19 has been described as the perfect epidemiological storm because of its structure and peculiar biology of infection, comparatively high contagion rates, lengthy incubation period, early and sustained virus load, the existence of asymptomatic or mildly symptomatic contagious carriers, long-term viral shedding, and propensity to progress toward “respiratory distress and death in up to 5–10% of cases” (Lippi et al., 2020, p. 2). Although the death rate of COVID-19 is lower than that of SARS or MERS, the total number of deaths from the disease thus far makes it the deadliest of the three coronaviruses (Lippi et al., 2020). Considering the hypothesized 2% death rate for COVID-19 and calculating similar infection rates as influenza, theoretically, the globe could witness a total of 52 million deaths before COVID-19 is no longer a pandemic (Wong et al., 2013; Lippi et al., 2020).

Although the world is far from this theorized potential number, as described earlier, the global death total as of the end of September was over one million people. Further, the total number of international cases, as of September 30, 2020, was approximately 34 million (Roser et al., 2020). For comparison’s sake, by April 28, the number of deaths from COVID-19 in the United States had surpassed the number killed in the Vietnam War (Strochlic et al., 2020). On June 3, the virus had killed more Americans than the Vietnam War, the Korean War, the Afghanistan War, and the Iraq War combined (Cuthbertson, 2020). Solely from descriptive statistics, it is clear that COVID-19 is a threat according to human security theory and biosecurity, a threat surpassing all combined wars that the United States has engaged in since World War II. But in what other parameters does the virus represent an existential security threat?

Although the full context of the disease will not be known for some time, already medical professionals and security experts have generated a plurality agreement on the existential crisis that COVID-19 represents (Bakir, 2020; Zumla & Niederman, 2020). As Gronvall (2020) notes, COVID-19 has been a catastrophe for the world and reflects poorly on the national security of the United States: “Insufficient quantities of supplies needed to protect the well and identify the sick have led to diminished, even negligible, situational awareness. Many lives have been lost. Military readiness has been degraded. The country’s economic power has dwindled” (p. 79). This section details how COVID-19 is a direct threat to international security and emphasizes the immediate danger to U.S. national security. The case study is not meant to be exhaustive but seeks only to demonstrate how COVID-19 threatens global security by focusing on the United States. Further research will present a comparative analysis of other international actors.

According to both human security theory and biosecurity, an ID is considered a threat based on scope, morbidity and mortality rates, and fear of contraction. The simple statistics on the number of cases globally and especially in the United States and the number of confirmed deaths are enough to satisfy this article’s claim that COVID-19 is a national and international security threat. In less than one year, the total number of confirmed deaths from the virus is over one million globally. For comparison’s sake, worldwide deaths associated with seasonal influenza vary each year from 290,000 to 650,000 (Freedman, 2020). By the end of 2020, the global death count for COVID-19 was 1.82 million (https://ourworldindata.org/covid-deaths). This surpasses even the most grievous years of seasonal flu deaths. But the damage to the international arena is not halted with the total number of confirmed infections and resulting deaths. The pandemic has had a gratuitous effect on the global economy as well.

China’s official assessment was that its gross domestic product (GDP) fell by 6.8% in the first quarter of 2020 compared with the first quarter of 2019; the Federal Reserve Bank of Atlanta estimated a 4% drop in GDP for the United States; France lost 6%. The numbers for economic stagnation and loss of economic activity are echoed by the Organisation for Economic Co-operation and Development: “Estimates range between –18% in China and –31% in Japan, with the US and France standing at around –25%” (Heisbourg, 2020, pg. 14). The International Monetary Fund forecasted a total –3% global GDP decline (Heisbourg, 2020). COVID-19’s impact on the U.S. stock market is unprecedented. The damage is more extensive than the 1918 Spanish influenza epidemic. Baker et al. (2020) write, “no previous infectious disease episode led to daily stock market swings that even remotely resemble the response in 2020 to COVID-19” (p. 746). In fact, COVID-19’s volatility rating exceeded the global financial of 2008 and nearly rivaled the effects of Black Monday in October 1987 (Baker et al., 2020 pg. 743). Banning travel to affected areas or denying entry to passengers from affected areas are somewhat effective measures for controlling the entrance of the virus (Chinazzi et al., 2020), but the social and economic impacts are far more significant because they affect all areas of business on a global scale: trade, agriculture, oil, manufacturing, finance, tourism, aviation, real estate, education, and entertainment. A new recession is feared, as well as financial ruin—as of this writing, it is not known what the real economic consequences of these measures will be (Petersen et al., 2020). According to the BBC, millions of people have been furloughed globally. Additionally, the BBC points out that as of June 2020, the proportion of people out of work in the United States hit 10.4%, signaling, according to the International Monetary Fund, “an end to a decade of expansion for one of the world’s largest economies” (Jones et al., 2020, para. 11).

The pandemic has also dealt a severe blow to the public health sector. As discussed earlier, public health and health security occur at the nexus of national security and health care. It is an area of interest for both paradigms discussed: human security theory and biosecurity. A health system includes all the institutions that work toward improving and restoring health in general (Palagyi et al., 2019). Health system responsiveness shows a state’s ability to take preventive measures, effective responses, and eventual recovery from a health emergency. This is measured by six core constructs, four focused on hardware, including surveillance, infrastructure and medical supplies, workforce, and communication mechanisms. Additionally, two are focused on software constructs, including governance and trust (Palagyi et al., 2019, p. 1850). Poor health system responsiveness has contributed to ineffective managing of the COVID-19 pandemic, as evidenced by personal protective equipment shortages, inadequate hospital capacities, and inefficient testing and case tracking, among other issues.

The COVID-19 pandemic has effectively changed how health care is handled, specifically the reorganization of resources. It has affected not only health care but also national security globally; it has changed the environment of public health and security (Chang & McAleer, 2020). New tools and shared global resources have been deployed to be utilized in disease prevention in hospitals and providing the general population with personal protection tools such as facemasks (Park et al., 2020; Wang et al., 2020). The rush to produce essential equipment for combating the virus and subsequent difficulties involved in getting them to medical professionals and the general public highlight a significant underlying issue: pandemic underpreparedness has added to the overall infection rate, death count, and expense associated with eventually defeating the virus. Additionally, it has also meant fewer resources being devoted to non-COVID-19-related health issues. The number of elective surgeries has decreased and, in some instances, stopped altogether, again threatening security according to human security theory.

General isolation mandates, restrictions for travel, country lockdowns, and changes in legal systems have had a remarkable impact on health care and national security worldwide. The quality of health care systems varies significantly among countries, demonstrating a lack of preparedness in many states, but especially in developing countries (Nicola et al., 2020). These measures have created changes in national and international law, causing many areas of everyday life to be affected, such as travel restrictions and border controls to prevent the spread of COVID-19. As described earlier, these restrictions have had severe economic consequences, but they have also served to separate and isolate families, adding to the fear and general angst of the pandemic. This, of course, has increased the threat according to human security theory. To limit the transmission across borders, many countries have implemented lockdown measures, including the full closure of businesses and airports and the complete closure of borders (Studdert & Hall, 2020). Many countries simply lack the power to effectively enforce bans on travel across borders, especially in impoverished and developing regions, which are already the most likely to suffer the most from the pandemic. This lack of control will almost certainly prolong the pandemic overall as active cases continue to spread across borders.

The pandemic has also disrupted public trust in government at all levels, including, in the United States, local, state, and federal authorities, the presidency, and the party system, and globally, the increase of conspiracy theories and nationalist and authoritarian movements in response to the pandemic have eroded government trust. Incidents such as the “Cummings event” serve to sow public discord between the population and the government elites (Fancourt et al., 2020, p. 464). In this particular case, Dominic Cummings (senior aide to the British prime minister) broke lockdown rules to travel to a family estate with his wife and child, who were suspected of being COVID-19 positive. This event was significant as it was the first by an official not to be followed by an apology and resignation (Fancourt et al., 2020). Cummings was widely condemned, and his transgressions eventually resulted in an apparent decline in the level of trust citizens held in the government. This is important because “public trust in the government’s ability to manage the pandemic is crucial as this trust underpins public attitudes and behaviors at a precarious tie for public health” (Fancourt et al., 2020, p. 464). As public trust in the government degrades, the situation is likely to degrade along with it as pandemic preventive procedures become more likely to be ignored, resulting in higher cases and death counts.

Issues related to compliance with stay-at-home orders have also been significantly politicized in the United Sates as partisan divides over prioritizing economic strength and stability versus public health have dominated congressional sessions and media coverage. Yet representatives of both parties have drawn public criticism for disobeying stay-at-home orders and virus guidelines. Speaker of the House Nancy Pelosi received a wave of criticism for visiting a San Francisco hair salon without a mask in September (Santucci, 2020). President Trump has been frequently criticized for refusing to wear a mask in public, most notably following his positive COVID-19 test result immediately after the first presidential debate on September 29; some polls indicated that Americans were more critical of the president’s handling of the COVID-19 crisis and his illness following his diagnosis (Pace et al., 2020). Lieutenant Governor Dan Patrick of Texas also came under fire in March for his controversial comments suggesting that the elderly would be willing to die to protect their children and grandchildren’s “economic future” (Knodel, 2020).

Stay-at-home orders were issued for almost all 50 U.S. states during the initial wave of COVID-19 cases, but the timing and severity of restrictions associated with these orders were affected by numerous factors, and significantly by partisanship (Murray & Murray, 2020). The politicization and subsequent polarization surrounding COVID-19 related guidelines have resulted in public condemnation, and in some cases, adoration, of officials who challenged or disobeyed restrictions. This has almost certainly contributed to the continued disregarding of public mask mandates and the flouting of COVID-19 restriction guidelines by American citizens who have lost confidence in public officials.

Further, social divisions are likely to be heightened by the pandemic and by the government’s response or perceived lack of response. Social tensions are likely to be elevated, especially in the United States. U.S. society is already experiencing divisions along racial lines; this could be amplified by the pandemic’s disproportionate impacts on underrepresented populations. A report published by the Council on Foreign Relations highlights, “Pandemics of infectious disease have disparate effects on elderly, low-income, marginalized, and other vulnerable populations within societies. In the current pandemic, infection and mortality rates have been highest among nursing home residents and Black, Indigenous, and Latinx communities, especially those inadequately served by the U.S. health-care system and bearing the brunt of socioeconomic disparities” (Burwell et al., 2020, p. 23). Public trust in the U.S. government could also be hampered by the lack of credible information delivered by the Trump administration, or, as some point out, the purposeful releasing of misinformation concerning the pandemic. As Hatcher (2020) evinces, “President Trump denied the danger of the virus and misled the public about the problem, the policies and procedures surrounding the problem, and the politics needed to solve the problem. This lack of transparency in communication … restricted the ability of the bureaucracy at all levels—federal, state, and local—to respond to the crisis” (p. 614).

According to Hatcher, this failure in communication had catastrophic effects, negatively influencing how citizens responded to the pandemic. First, President Trump’s communication encouraged protestors in several states to disregard social distancing and stay-at-home guidelines. Second, the president downplayed the seriousness of the pandemic repeatedly, often treating it as less of a threat than the seasonal flu. And third, the president spread confusion by not validating the Centers for Disease Control and Prevention’s guidance regarding the benefits of wearing cloth masks in public (Hatcher, 2020). The poor communication from President Trump, if not negligent, hampered the capability of public health professionals to remedy the disease and to safeguard the public against it. As Hatcher (2020) writes, “The spreading of misleading information, through inciting social pressures, hampers public health campaigns. Public health campaigns are not able to break through the misinformation to promote evidence-based behaviors, removing the efficacy of social pressure efforts” (p. 615).

COVID-19 has also had a detrimental impact on the readiness and preparedness of militaries worldwide and has dramatically hindered the U.S. armed forces (Gostin & Wiley, 2020). The USS *Theodore Roosevelt* aircraft carrier was effectively isolated and quarantined while docked in Guam because of rapidly spreading infection rates onboard (Gronvall, 2020). Of the 4,085 sailors taken off the ship, 736 tested positive for COVID-19, with 500 being symptomatic for a median duration of 7 days (Alvarado et al., 2020). States have acted with a focus on controlling the spread of COVID-19 among soldiers, as an outbreak could impede the operational capacity of the armed forces by reducing its workforce. This would negatively impact the ability of states to defend themselves and participate in creating security in the international arena. Per the U.S. Department of Defense’s (2020) current count, as of October 21, 2020, 76,484 service members have been infected with 1,605 hospitalizations, 52,173 recoveries, and 102 deaths.

Throughout the U.S. armed forces, training formats have been altered to accommodate social distancing when possible (Military Health System, 2020a), recruitment efforts have been changed and diminished (Green, 2020), military hospitals and other operational assets have been re-tasked to fight the virus (Lopez, 2020; Military Health System, 2020b), and the number of military preparedness exercises has decreased (The Cipher Brief, 2020). The Defense Department also issued an order to cease all travel in March as one of its first significant steps toward curbing the spread of the virus among military personnel. This order froze all military personnel and their families, domestic and abroad, until late June (Military One Source, 2020). The U.S. military was poised to deploy in several capacities throughout the crisis, including “coordinating the movement of supplies, setting up field hospitals and temporary shelters and stepping in to fill gaps in health care if first responders fell ill” (Young & Raphaelson, 2020, para. 5). Several U.S. states activated National Guard troops to assist in maintaining testing sites for COVID-19, demonstrating the potential for an effective military to combat pandemics (Young & Raphaelson, 2020). Other states outside the United States activated their militaries to ensure observance of curfews.

The Israeli Defense Force (IDF) shifted military meetings to primarily video calling and issued curfews affecting both military members and civilians “to minimize exposure between civilians and military personnel and to decrease the amount of people who used public transportation on a national scale” (Segal et al., 2020, pg. e1626). Initial studies indicate that the IDF’s strategy resulted in an effective risk management policy that did not severely limit workforce capacity (Segal et al., 2020). However, some military efforts to enforce lockdowns have been less successful, particularly in Africa. Allegations of abuse leveled at soldiers and police enforcing lockdowns have been rampant. In Kenya, the armed forces were forced to issue a public apology after assaulting a crowd near a train station with tear gas, clubs, and whips to disperse them. Simultaneously, a police officer in South Africa was arrested for killing a man and wounding three children who stepped out onto their porch after curfew (Dyer, 2020). Security forces in Africa typically garner little, if any, public trust, and videos of military personnel and police officers assaulting lockdown violators make matters worse (Dyer, 2020). Surveys indicate only 39% of respondents in South Africa felt that the army “often” or “always” behaved professionally during lockdown enforcement procedures (Isbell, 2020, pg. 12).

There is also potential for great power unrest and geopolitical revisionism. Heisbourg (2020) notes that COVID-19 is likely to increase domestic instability in the United States, and if this happens, President Trump’s America First policy is likely to morph into broad-based isolationism (p. 17). Depending on America’s response to the pandemic and potential increasing domestic instability, other nation-state actors may see a window of vulnerability and move to increase their international standing. This is especially true of China and Russia. Combined with global discontent with President Trump’s foreign policy, China may try to strengthen its international standing vis-à-vis the United States. Moreover, the economic downturn could spell total collapse for the European Union. Using worse-case extremism, Heisbourg (2020) posits, “Blandishments, corruption and pressure from China and Russia further splits the Union, while Trump makes the presence of US forces in Europe contingent on the kind of deal he has contemplated imposing on South Korea. The EU ends up as a latter-day version of the Holy Roman Empire in its sunset years. France and the UK eventually lose the means to sustain their current rank as world-class diplomatic and military powers, and Germany never gets there” (p. 19). Besides great power rivalry, the international arena has experienced an increase in threats from terrorism (as a result of the pandemic) and cyber-based attacks. These cyberattacks have both broad kinetic and information-related impacts.^3^

Concerning terrorism, there have been increased calls from terrorist organizations for attacks on U.K. hospitals and other vulnerable places during the coronavirus outbreak, according to the U.K.-based *Independent* newspaper: “Protective security advice is being distributed to [National Health System] trusts by counterterror officers, amid warnings that extremists are exploiting the pandemic to radicalize new recruits” (Dearden, 2020, para. 2). Additionally, ISIS and al-Qaeda have used the pandemic as an operational tool, calling the pandemic a “solider of Allah” and stating that the virus is a divine punishment for nonbelievers (Danvers, 2020, para. 7). There is speculation that the virus could be a “blueprint” for future bioterrorist plots (Means, 2020, para. 14). Cyberattacks have also increased during the pandemic. For instance, remote desktop protocol attacks grew 400% in March and April 2020. Email scams related to COVID-19 surged 667% in March 2020, and there was a 2000% increase in malicious files with “Zoom” in the name (Gewirtz, 2020, para. 17). This increase in cybercrime can reasonably be associated with the stay-at-home protocol created as a result of COVID-19. Disinformation operations concerning the origin of the virus and the conspiracy theory of U.S. military involvement in its generation can be traced to January 2020 (Molter & Webster, 2020).

Additionally, Russian bots have been used to promote two disinformation theories related to the epidemic: the intentional creation and engineering of COVID-19 in a laboratory as a biological weapon, and the notion that the pandemic is being used to conceal and distract the general populace from the harmful effects of new 5G towers. Bots are responsible for 70% of COVID-19 information on Twitter (Marineau, 2020). The consequences for human health and safety and the effects on hospital infrastructure, the national and global economy, and militaries worldwide demonstrate clearly that COVID-19 is a security threat according to both the human security theory and biosecurity frameworks. It is a threat to the health and well-being of citizens and to the global economy, and it has threatened the capabilities of armed services worldwide. Additionally, the presence of COVID-19 has amplified geopolitical tensions, the threat from terrorists seeking to take advantage of the opportunity during the pandemic, and cyberattacks. However, if the pandemic continues to surge through the winter of 2020–2021, the potential for even greater worldwide catastrophe exists, amplifying the call to consider COVID-19 a threat to national security.

## Conclusion

The politicization of the epidemic around the world has crippled otherwise capable states’ ability to curb the virus. States continue to fail to reach the core capacities emphasized by Davies (2013). COVID-19 has not only reemphasized Price-Smith’s (2008) point that the global system was weakly prepared for a threat of this variety, it has, more troublingly, unveiled a potential *decline* in pandemic preparedness. Pandemics have not simply remained a low priority to policymakers, their priority has decreased. Commitments to health security are no longer just impeded by broken health systems and obstacles to their repair. Rather, health security has become politicized and commitments from some states to its cause have become mere rhetoric, as Davies (2013) feared they may. In essence, this article is important because it highlights more than a stalling of progress. It points out that preparedness prospects have gotten worse, and the price of that decline may be the United States’ reputation.

Given the demonstrated short-term effects of this crisis, it is imperative that global security discussions and policymaking shift their perspective on ID for the future. ID should henceforth be addressed as a high-level threat. COVID-19 has sent even the world’s most powerful states, in terms of both military strength and economics, scrambling to address the crisis. The international community has hitherto underestimated the threat posed by pandemics and, as a result, was woefully unprepared to confront the danger or mitigate the scale of the damage. This is the worst-case scenario in the context of security threats for many states: an enemy that transcends borders, presents an existential threat, and cannot be stopped by conventional military means. Without seeing the full-scale of the effects of the crisis, it is impossible to be sure that the worst is not yet to come.

All of this combines to potentially create a perfect storm in which states face internal security threats from fear, panic, riots, rebellion, criminal activity, and terrorism, on top of the actual biological effects of the disease, leading to international threats. Even if the United States is not directly threatened by global conflict or domestic insecurity because of COVID-19, the strategic implications of geopolitical repositioning increase potential instability. For instance, what if North Korea experiences a catastrophic influx of COVID-19 rates, increasing its medical, food, and humanitarian needs? What if the North Koreans cannot obtain these needs diplomatically and resort to the coercion of South Korea or other state actors? Further, if Russia experiences a decline in its military readiness, it may act preventively in Belarus, Ukraine, Georgia, or other powers within its sphere of influence and strike first to increase leverage over these actors before the window of opportunity closes. This threat applies now more than ever to cyberspace and artificial intelligence as well. Perhaps Iran perceives the United States as unstable or losing power and prestige on the international stage because of COVID-19. Or, in combination with the widespread perception that the United States mishandled the virus and current racial tensions and unrest resulting from perceptions of police brutality, China may increase its cybercriminal activities, targeting large U.S. businesses to steal millions of assets and perhaps technology when they believe that U.S. federal agencies are preoccupied?

It is already acknowledged that the United States is facing severe cyberattacks by its strategic adversaries to sow discord and division through information warfare and attacks on critical infrastructure. There is ample evidence of the geopolitical tensions that COVID-19 has heightened. This is attributed to increasing nationalism (Heisbourg, 2020, p. 12) and authoritarianism (Simon, 2020); it has also had global repercussions for internal discord. As Heisbourg (2020, p. 12) evinces, “Catalonia and Madrid have clashed over which jurisdiction is in charge of confinement rules. In the US and Belgium, disputes have arisen between federal and state authorities over the allocation of personal protective equipment. Sicilians have not always welcomed Lombardian ‘corona-refugees.’”

The high population density in urban settings allows for the rapid spread of ID. Depending on the death ratio and speed of contagion, there could be resulting high death rates, which could lead to panic and fear, violence, and looting in an attempt to acquire scarce lifesaving resources such as medicine and treatment as well as necessities such as food and water. Combined with the possibility of disruption in food supply and logistics, damage to critical infrastructure such water-treatment plants, and a rise in unemployment, homelessness, and so on, this could lead to an increase in internally displaced persons—who, wandering, would continue to spread the disease, amplifying all of the above. If internationally displaced persons continue to seek refugee status, large gatherings or refugee camps could provoke more violence and, in the vicinity of multiple borders, cause cross-national conflict. Further, the spread of international conflict exacerbates the situation even more by spreading ID further. In this manner, it has a cyclic nature: ID could create tensions that cause warfare, thereby increasing the spread of more ID. Koblentz (2010) summarizes the situation aptly: “Internal conflicts facilitate disease outbreaks by destroying a nation’s medical and public health infrastructure, generating large volumes of displaced persons who lack adequate food, shelter, sanitation, and medical care, and by impeding assistance from international public health and humanitarian organizations” (p. 103).

Heisbourg notes that the pandemic has given new salience to borders, even in government organizations where borders are relatively porous. For instance, the scholar notes that Germany and France are filtering incoming traffic across their borders because of the pandemic; China has sealed off Hubei Province from the rest of the country; and in the United States, President Trump suspended green cards granting permanent legal residence to certain immigrants during the pandemic (Heisbourg, 2020). March 17, 2020, marked the first time in the history of the European Union that all of its external borders were closed in hopes of containing the virus (Linka et al., 2020). States across the Middle East and Africa, which were likely least prepared to combat the virus, have resorted to closing borders and quarantining returning citizens (Da’ar et al., 2020). However, these measures are likely too little, too late. As McKibbin and Fernando (2020) argue, “The idea that any country can be an island in an integrated global economy has been proven wrong by the latest outbreak of COVID-19” (p. 25 ).

The damage wrought by COVID-19 on the world, and particularly the United States, is exceptionally troubling moving forward. Even when the crisis draws to a close, its impacts and the weaknesses it exposed will not be forgotten. The potential for devastating bioterror and biological attacks has been heightened by COVID-19, as it is now evident to the world, including America’s enemies, that the United States is woefully underprepared for threats of this variety. By underestimating the dangers posed by pandemic diseases and focusing on other, more conventional high-politics issues such as international war, America has demonstrated that it is vulnerable to precisely the kind of damage it is now enduring from COVID-19 (Bearman et al., 2020). As Gronvall (2020) notes, strategic adversaries may conclude from the U.S. response to COVID-19 and the harm it has caused to military readiness and the economy that the same effects could be replicated by a biological attack (p. 81). The long-term damage from this crisis will likely extend beyond the death toll and economic decline. America’s reputation has been called into question. Concepts such as “American exceptionalism” are in doubt for the first time in decades as America’s enemies and allies celebrate and mourn what some perceive as the superpower’s decline (Haiphong, 2020, p. 200). The United States must take decisive action to prepare itself for the next crisis and reassert itself as a global leader by setting an example for other countries on pandemic preparedness.

Biological threats like COVID-19 are unique in that once the threat has emerged, it is already too late to combat it properly. If the United States is to curb the next biological menace, it must prepare to fight it with the same commitment it would dedicate to preparing for international conflict. As demonstrated by the COVID-19 crisis, biological threats must henceforth be considered an issue of high politics with potentially existential consequences. Officials have warned for years that the threat posed by respiratory viruses like SARS, MERS, and now COVID-19 will only increase as new viruses continue to emerge (Al-Tawfiq et al., 2014; Gillem-Ross and Subbarao, 2006; Hayden, 2006). A useful answer to this threat is to return to the pandemic preparedness mentality and the strategies proposed by President George W. Bush and adapted and improved on by President Obama. The 2005 National Strategy for Pandemic Influenza expressed the necessary mentality well:

Preparing for a pandemic requires the leveraging of all instruments of national power, and coordinated action by all segments of government and society. Influenza viruses do not respect the distinctions of race, sex, age, profession or nationality, and are not constrained by geographic boundaries. The next pandemic is likely to come in waves, each lasting months, and pass through communities across the nation and world … This makes a pandemic a unique circumstance necessitating a strategy that extends well beyond health and medical boundaries, to include the sustainment of critical infrastructure, private-sector activities, the movement of goods and services across the world, and economic and security considerations. (Homeland Security Council, 2005, p. 2)

This approach to pandemic preparedness will be of paramount importance going forward to ensure that the United States will be better prepared to confront the challenges posed by pandemics. In light of the threat posed by pandemic flu and the terrorist anthrax attacks in 2001, the Bush administration’s enactment of the Pandemic and All-Hazards Preparedness Act in 2006 focused on improving biological threat preparedness efforts by “centralizing federal responsibilities, requiring state-based accountability, proposing new national surveillance methods, addressing surge capacity, and facilitating the development of vaccines and other scarce resources” (Hodge et al., 2007, p. 1708). While this act was controversial at the time, its efforts would have undoubtedly proved beneficial to current U.S. efforts to combat COVID-19 and could still be beneficial in the future should the United States face another pandemic or the threat of a biological attack.

The Obama administration was faced with its own pandemic threat in the form of an Ebola outbreak. The situation could have spiraled rapidly following the president’s announcement of three confirmed U.S. cases in October 2014, but decisive action on the part of the administration, including the creation of expert “SWAT teams” ready to be deployed to any hospital with an active case and previous preparedness efforts, halted the virus’s spread in the United States (McCarthy, 2014, p. g6333). America further emerged as a leader in the fight against the disease’s spread across Africa, with the UN Security Council officially declaring the outbreak a “threat to international peace and security” on September 18, 2014 (Butler, 2014, p. 469). The United States subsequently deployed 3,000 military personnel to West Africa, where the disease was most prevalent, and dedicated $750 million to “support civilian efforts” on the ground in combating the virus (Butler, 2014, p. 469). While the death toll was high, the outbreak was mostly contained and prevented from becoming a global pandemic.

SARS-CoV-2 is a novel virus. As such, the dynamically changing scientific evidence creates a needed understanding of variation in evolving information and facts. Information regarding the virus must be made publicly available as quickly as possible to counter its spread. The inherent caveat of this strategy is that this information is often based on short-term observations that have not been verified over the usual long testing periods, and therefore its accuracy is limited. Contradictions between initial reports and subsequent data thus become more likely (Phelan et al., 2020). This creates challenges with standardizing protocols and international policy, thus creating an opportunity to create politically divisive narratives that feed political instability (Price-Smith, 2008). This can become especially problematic in countries undergoing elections. Another international security challenge associated with a lack of standards is the difficulty of balancing economic opportunities and public health needs (Price-Smith, 2008). Some countries and regions may choose to loosen restrictions to lessen the economic damage associated with pandemic control measures. This can create threats to the national security of bordering and airspace-connected countries which are still prioritizing public health. Globalization has rendered all states vulnerable to the consequences of one state’s failure to contain a threat of this nature (Davies, 2013).

In summary, this article suggests that the United States implement the following actions to contain ID and prevent it from further threatening national security. Perhaps most importantly, ID should be considered a high-level security threat moving forward and should be included in the high-politics discourse.

Internationally, the United States should further call for other states to take a similar posture when considering ID. Subsequently, rejoining the WHO and other UN-based international health regimes should be a high priority, as the United States will need to demonstrate its own commitment to this cause. The United States must lead this new front against ID by example, both to reestablish its own credibility and to unite its allies against ID threats. The failure of any one state to contain a pandemic threat is nigh insurance that others will suffer the ramifications, as COVID-19 has demonstrated.

Domestically, the United States should establish systems of early ID threat notification and assume a posture prioritizing transparency and accuracy regarding information related to outbreaks of any new ID threats. Reinstating successful strategies devised by previous administrations, such as the ID SWAT teams created by the Obama administration and the respirator stockpile established by the George W. Bush administration, should also be of high priority. The establishment of more organized medical surveillance systems such as the U.S. military’s Defense Medical Surveillance System might be a useful tool for early detection of new ID threats (Rubertone & Brundage, 2002). These systems routinely collect and analyze samples from a population for the purposes of “detecting, characterizing, and countering threats to the health, fitness, well-being, and performance” of that population and would require careful organization and focused guidelines to protect patient privacy, but might prove instrumental in curbing outbreaks during their early stages (Rubertone & Brundage, 2002, p. 1900). Incorporating these strategies into the U.S. national security agenda will benefit the United States and the global community as a whole by reestablishing strong global leadership and faith in vital international organizations to help avoid another chaotic pandemic response (Johnson, 2020). Furthermore, cracks in the complex global system exposed by ID threats might be repaired (Benatar, 2002).

The gravity of biological threats and the importance of adequately preparing for them is not new to the world. U.S. preparedness efforts have waivered in recent years, setting the stage for COVID-19’s extensive damage. Future pandemic preparedness policies should, above all, treat biological threats as having the potential to be existential in scale, build on the successful efforts of past administrations, and expand domestic preparedness. As Barry (2010, p. 10) notes, “It is the nature of the influenza virus to cause pandemics. There have been at least 11 in the last 300 years, and there will certainly be another one, and one after that, and another after that.” The issue of respiratory pandemics and potential biological attacks will remain of grave importance and should be grounded in discussions of high politics going forward. Examining ID through both human security theory and biosecurity frameworks helps situate pandemics within the language and policy of national security.

## Footnotes

1 See the Appendix.

2 For background and primers on biosecurity and biodefense, see Hoyt and Brooks (2003–2004), Koblentz (2003–2004), and Tucker (2006).

3 Special thanks to Sarah Rees, director of the Cyber Workforce Academy, Georgia Cyber Center, for this information.
